# Recent advancements in the application of artificial intelligence-based approaches for screening, diagnosis, prognosis and treatment of cervical cancer

**DOI:** 10.3389/or.2026.1760156

**Published:** 2026-05-11

**Authors:** SubbaRao V. Tulimilli, Medha Karnik, Sam Cockroft, Anjali Devi S. Bettadapura, Suma M. Nataraj, Habeeb Shaik Mohideen, Sinisa Dovat, Arati Sharma, SubbaRao V. Madhunapantula

**Affiliations:** 1 Center of Excellence in Molecular Biology and Regenerative Medicine (CEMR) Laboratory (A DST-FIST Supported Center and ICMR-Collaborating Center of Excellence), Department of Biochemistry (A DST-FIST Supported Department), JSS Medical College, JSS Academy of Higher Education and Research (JSS AHER), Mysuru, Karnataka, India; 2 Department of Molecular and Precision Medicine (SC, AS), Department of Pediatrics (SD), Center for Cannabis and Natural Product Pharmaceuticals (CCNPP) (AS), Penn State Cancer Institute, Pennsylvania State University College of Medicine, Hershey, PA, United States; 3 Bioinformatics and Integrative Omics Laboratory, Department of Genetic Engineering, School of Bioengineering, College of Engineering and Technology, SRM Institute of Science and Technology (formerly SRM University) Kattankulathur, Chengalpattu, India; 4 Special Interest Group in Cancer Biology and Cancer Stem Cells (SIG-CBCSC), JSS Medical College, JSS Academy of Higher Education and Research (JSS AHER), Mysuru, Karnataka, India

**Keywords:** artificial intelligence, cervical cancer, deep learning, diagnosis, machine learning, prognosis, screening, treatment

## Abstract

Cervical cancer (CC) is the fourth most common type of cancer among women. Majority of CC cases (84%–90%) have been reported in low- and middle-income countries (LMICs). Persistent infection with high-risk human papilloma virus (HR-HPV) subtypes is responsible for >90% of the cervical cancers. CC is preventable by timely vaccination with HPV vaccine. Early diagnosis, treatment and disease recurrence prediction markers play a significant role in improving the patient outcome, yet LMICs are seeing a continuous increase in CC cases. Lack of sensitive self-screening technologies, effective diagnostic methods and accessible treatment options are predominantly contributing to this increase. Recent advances in artificial intelligence (AI), particularly machine learning (ML) and deep learning (DL) methods have revolutionized CC diagnosis, prognosis, and treatment agent selection by (a) enhancing the accuracy of screening and diagnostic methods; (b) assisting the clinicians in predicting various prognostic factors such as lymph node metastasis, treatment response, survival outcome, postoperative risk factors; and (c) providing dose prediction, treatment planning, segmentation of target volume and organ at risk. AI algorithms can analyze complex datasets, including medical images, patient data, and genetic information, to identify patterns and predict outcomes that might have not been considered by traditional methods. AI-based analysis provides more accurate diagnosis and improved risk stratification in a very short duration, while helping in the design of tailored treatment strategies. In this article, we aim to provide an overview of emerging ML and DL methods and comprehensively evaluate the role of AI-based approaches in the screening, diagnosis, prognosis and treatment of CC. Finally, we discuss challenges and limitations associated with the use of AI models in CC screening, diagnosis, prognosis and treatment. We have also focused on emerging AI models that can be applied in CC research and treatment to overcome the current challenges and limitations.

## 1 Introduction

**FIGURE 1:**
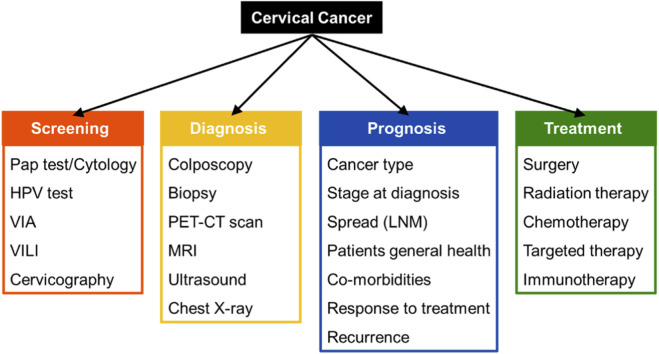
Methods used for screening, diagnosis, prognosis and treatment of cervical cancer.

Cervical cancer (CC) is the fourth most common cancer in women globally, with 2022 data showing 662,301 new cases and 348,709 deaths (1, 2). Persistent, high-risk human papillomavirus (HR-HPV) infection causes over 99% of CCs, with HPV types 16 and 18 alone responsible for approximately 70% of cases (3, 4). Despite being highly preventable via vaccination, cervical cancer remains a leading cause of mortality in low- and middle-income countries (LMICs) (5). Secondary prevention strategies include early screening, accurate diagnosis and effective treatment of precancerous lesions (6, 7). Various methods used for screening, diagnosis, prognosis and treatment of CC are depicted in Figure 1.

The World Health Organization (WHO) has set a 90-70-90 strategy for the elimination of CC by the year 2030 (7). It aims for the vaccination of 90% girls under the age of 15 years by HPV vaccine (Gardasil or Cervavac), screening of 70% of women aged between 35–45 years, and treatment of 90% of those diagnosed with CC. Technological advancements have been contributing immensely to achieve this goal by providing more accurate strategies for effective diagnosis and better treatment decisions. For instance, subsets of artificial intelligence (AI) have been extensively used for early diagnosis, better assessment of prognosis and treatment outcome prediction of CC, among other cancers (8–10). The application of several AI models in CC screening, diagnosis, prognosis, and treatment has been reported in recent literature, however, very few studies have comprehensively evaluated the merits, shortcomings and necessary precautionary measures to be implemented while using AI-based approaches. This shortfall motivated us to write this comprehensive review article, in which we have thoroughly studied and summarized various AI models used in the screening, diagnosis, prognosis and treatment of CC. Beyond analyzing current AI limitations, we explored how emerging models can tackle unresolved challenges in CC research.

## 2 Artificial intelligence (AI) and its subtypes

Artificial intelligence (AI) is the simulation of human cognitive processes by machines, enabling them to learn, reason, solve problems, and make decisions (11). Machine learning (ML) is an AI subset, which uses data-driven algorithms to train systems to learn, adapt, and make decisions independently without explicit programming (12). Deep learning (DL), a specialized branch of machine learning, employs multi-layered artificial neural networks to simulate the human brain’s cognitive functions for analyzing data and recognizing complex patterns (13).

### 2.1 Machine learning (ML)

In contrast to conventional programming, where a computer receives explicit instructions to execute various tasks, ML algorithms operate differently by analyzing data, identifying patterns, and making predictions or decisions without relying on pre-defined instructions (12). ML models are categorized into four primary types based on the specific learning approach used: supervised learning, unsupervised learning, semi-supervised learning, and reinforcement learning (13).

In supervised learning, algorithms learn from labelled data (where input and output relationships are known) through tasks including classification and regression (12, 13). For instance, patient health records and cytological images from Pap test results have been used in identifying specific patterns, which facilitates in accurate early diagnosis and staging (14). Among various supervised learning AI models, the most widely used models are (a) Random Forest (RF); (b) Decision Tree (DT); (c) Multilayer Perception Neural Networks (MLP-NN); and (d) Adaptive Boosting are reported (15). These supervised learning algorithms have been reported to achieve an accuracy of more than 90% in the disease diagnosis. Despite these advantages with supervised learning algorithms certain limitations still persist that include (a) involvement of a trained clinician to capture the images accurately; (b) lack of validation in real-world scenario and (c) lack of a system to make decisions based on blood biomarkers. Furthermore, the advancements are needed to upgrade the supervised learning models to consider the including the factors other than HPV infection, which are involved in the pathogenesis of CC. Currently, these limitations are being addressed by rapidly evolving AI systems (15).

Unsupervised learning analyzes unlabeled data to uncover hidden patterns or groupings thereby assist in identifying new cancer types and patient subgroups (12, 13). This method also helps in developing novel and specific biomarker panels, which assists in early monitoring and treatment effects. Among various applications of unsupervised learning, the analysis of complex images (by Fluorescence Lifetime Imaging Microscopy - FLIM) has gained more popularity due to its ability to produce highly specific (100%) and sensitive (∼91%) results that helps in detecting the cervical cancers with very high accuracy (16). Outlier detection, data dimensionality reduction and clustering, and sub-grouping are few other examples of unsupervised learning methods widely used in the cervical cancer research (17). UCI ML repository and Kaggle’s CC data sets are being widely used to train and test the unsupervised learning models (18). For additional details pertaining to these publicly available databases the readers may visit respective websites. Despite various advantages, the unsupervised learning models still suffer from various limitations that include (a) clinical validation; (b) data quality assessment and (c) interpretation of results. Therefore, future studies should focus on addressing these limitations for better diagnosis and clinical applicability.

Semi-supervised learning (SSL) blends both approaches by combining a small amount of labeled data with a large amount of unlabeled data (19). In CC, SSL helps in reducing the cost of diagnostic tests such as Pap test and colposcopic image analysis. Moreover, results obtained by SSL models exhibit high specificity and accuracy even with 50% labelled data sets (20). Despite these benefits, SSL suffers from noisy labels, diagnostic costs and real-world applicability. Further advancements are needed to address of these limitations.

**FIGURE 2:**
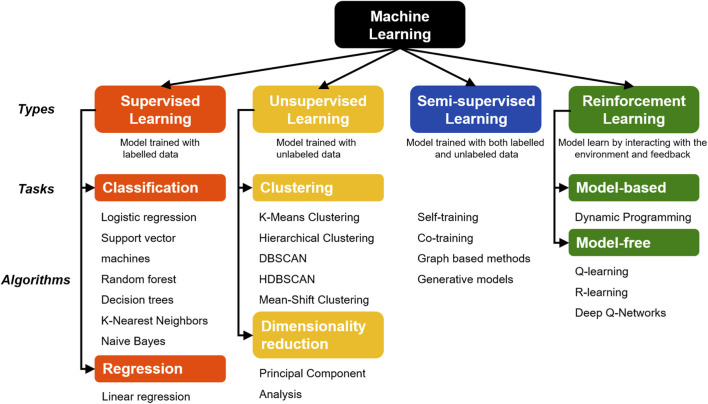
Classification of machine learning algorithms.

Reinforcement learning (RL) trains agents through environment interaction and feedback (21). RL focuses on improving diagnostic accuracy and assists in optimizing treatment planning (22). In addition, RL is being used in addressing data imbalance by focusing more on target cell population rather than considering the non-cancerous normal cells. Furthermore, RL helps in treatment simulation and radiation therapy optimization (23). However, implementation of RL in clinical practice is still in its incipient stages, hence, warrants further investigations. Classification of machine learning algorithms is depicted in Figure 2.

### 2.2 Deep learning (DL)

**FIGURE 3:**
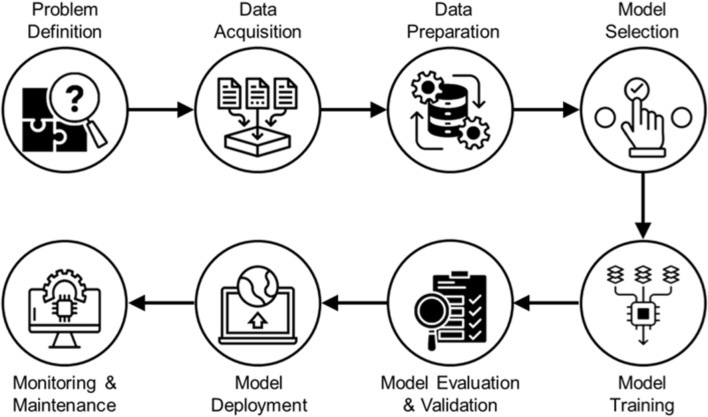
Schematic representation of steps involved in developing AI models.

Deep Learning is an advanced ML technique that uses deep neural networks to automatically learn high-level patterns from large datasets, enabling complex automation and predictive modeling (13, 24). The most common DL models and architectures used in healthcare are convolutional neural networks (CNNs), recurrent neural networks (RNN), residual networks (ResNet) and densely connected convolutional networks (DenseNet), autoencoders, generative adversarial networks (GANs), electronic health records (EHRs), natural language processing (NLP) (25, 26). Various steps involved in the development of AI models are shown in Figure 3.

## 3 AI in cervical cancer screening and diagnosis

Pap test or cytology (both conventional and liquid-based), HPV test, HPV/Pap co-test, visual inspection with acetic acid (VIA), visual inspection with Lugol’s iodine (VILI) and cervicography are the most common tests used for screening individuals for CC. In addition, techniques such as colposcopy and biopsy examination have also been used for the diagnosis of CC (27–30).

### 3.1 AI models for interpreting VIA and cervicography results

VIA is viewed as a viable alternative to the Pap smear test for CC screening in low-resource settings; however, the interpretation of the data primarily depends on the experience of examiner. Harsono et al. (31) developed an AI-based Android application to detect cervical pre-cancerous lesions using VIA test results. Results of this study indicate that the model attained a specificity of 96.4% and an accuracy of 93.8% in the VIA test (31).

In lieu of immediate visual inspection by a physician, cervicography is another common screening method preferred in LMIC. A novel DL architecture, “CervixNet,” has been proposed for classifying cervigram images into healthy and abnormal categories. CervixNet’s architecture was evaluated for sensitivity, specificity and accuracy in comparison to two cervigram databases, achieving more than 98.69% accuracy across all six measures (32).

### 3.2 AI for interpreting Pap test or cytology

AI efforts have extended to Pap test or cytology, the most common screening method for CC. (33) a DL-based liquid cytology model was developed using whole cell cytology slides to detect CIN2 (33). The study reported that the DL model alone was effective in detecting CIN2 and the model’s sensitivity matched with senior cytopathologists in community-based screening (33).

Beyond detection, AI can be used to aid CC cell classification. (34) a comparison study of sixteen pre-trained models was conducted for CC classification, utilizing single-cell Pap smear images sourced from the Herlev and Sipakmed datasets (34). Of the sixteen deep transfer learning (DTL) models, ResNet50, VGG16 and DenseNet121 achieved accuracies greater than 95% in at least one multi-class classification, indicating that DTL models are suitable for automating CC screening. DenseNet121 attained an accuracy of 97.65% for 3-class classification, while VGG16 obtained an accuracy of 99.95% for 2-class and 5-class classification based on the Sipakmed dataset (34).

Many novel systems sequentially integrate several AI methods into a multi-step pipeline. (35) classified CC using a novel approach that utilized four different DL in consecutive stages of this research. The proposed approach achieved an average accuracy of 99.6% for 3-class classification and 99.65% for 5-class classification on the SipakMed dataset (35). In a similar study, (36) introduced a novel classification framework that sequentially deployed Neural Feature Extractor (NFE) and AutoInt model before testing different ML classifiers. From this DL-based image classification, authors reported that simpler models exhibit faster prediction times, while more complex models provide higher accuracy. For instance, K-nearest neighbors reached an accuracy of 99.96%, while the light gradient boosting model achieved 99.92% accuracy (36). Similarly, (37) presented a novel DL-based decision support system aimed at identifying CC from the images of liquid-based cytology Pap smears (37). The study reported that this approach achieves a classification accuracy of 99.9% and sensitivity of 99.8% to detect the stages of CC (37).

DL-based ensemble models have also been used to produce more accurate and robust classification predictions than single models alone. In contrast to pipeline models, this technique pools results from individual models that run in parallel. (38) utilized the ensemble model for 5-class classification of cervical squamous found on Pap smear images from the SipakMed database. The ensemble technique combined CNN, AlexNet and SqueezeNet and achieved 94% accuracy, surpassing the performance of each individual model alone (38).

**FIGURE 4:**
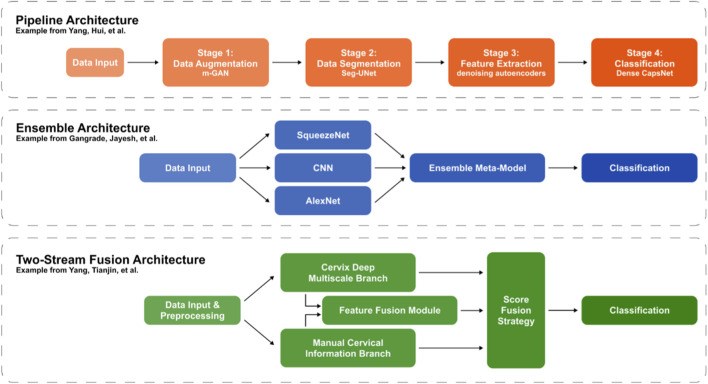
Model architectures for cervical cancer classification.

A two-stream decision fusion network was developed (39) to classify cervical Pap smear images. In contrast to pipeline and ensemble models, this two-stream technique combines individual streams into one cohesive model. This study’s technique demonstrated superior performance compared to state-of-the-art cervical cell classification models (39). This study’s two-stream architecture was compared to pipeline and ensemble model examples and is depicted in Figure 4.

Conventional and liquid-based Pap test or cytology can be used to generate whole slide images (WSIs). WSIs generate a high volume of data making AI a useful tool for analysis and classification. (40) developed a novel Compound Scaling Hypergraph Neural Network model (CSHG-CervixNet) for robust classification of CC subtypes using cytology WSIs and reported efficient and accurate classification of CC subtypes (40). Separately, Austin et al. (41) created a CNN-based approach to classify Pap smear WSI as either abnormal or healthy cells. The approach accurately distinguished cells with an accuracy of 97.55% for 5-cell classification using SipakMed dataset (41).

(42) proposed Cervical Cell Copy-Pasting (C^3^P), a self-supervised learning-based augmentation strategy to enhance data from unlabeled HPV^+ve^ cervical cytology images in low resource settings (42). Models trained on C^3^P outperformed other pre-trained models including classification of WSIs using established multiple instance learning (MIL) methods (42).

Federated learning builds a robust deep-learning model by utilising a variety of datasets from multiple collaborators. Federated learning enables model training on decentralized data while preserving privacy, as it keeps sensitive information local. Devices perform local training, and send only model updates to a central server, which aggregates these updates to improve a global model and returns it, creating a collaborative learning process. Data privacy is one of the significant challenges with the use of AI. (43), developed a novel CNN-based FL architecture to overcome data sharing challenges and achieve accurate image classification (43).

Reinforcement learning (RL) trains algorithms by interacting with the environment and receiving feedback in the form of rewards or penalties. In order to improve the efficiency and precision of CC screenings, (44) presented RL-Cervix.Net, a hybrid model, which combines RL and CNN technologies. A classification accuracy of 99.98% was achieved by RL-Cervix.Net in identifying atypical cells indicative of cervical lesions. The model addressed variability and complexities present in cytological images and showed better accuracy and interpretability than existing methods (44).

### 3.3 AI for interpreting histopathology

More complex supervised AI pipelines have also been studied with WSIs. (45) developed a DL pipeline, Silva3-AI, for the automatic analysis of WSI-based histopathological images, enabling accurate classification of HPV-associated endocervical adenocarcinomas through stromal invasion pattern identification. The Silva3-AI model exhibited improved interpretability, high accuracy and robustness (45).

### 3.4 AI for interpreting colposcopy results

Colposcopy plays an important role in the screening and diagnosis of CC (46). Since cervical transforming zone is regarded as one of the important factors for classifying colposcopy findings, (47) developed a DL-based multiscale feature fusion classification network for automatic classification of colposcopy images based on cervical transformation zone (47). The proposed method achieved accuracy of 88.49% and sensitivity of 90.12%, 85.95% and 89.45% to type 1, 2 and 3 respectively (47).

(48) introduced a novel “leveraging swin transformer with ensemble of DL model for CC screening (LSTEDL-CCS)” technique for the detection and classification of colposcopy images. The proposed LSTEDL-CCS performs feature extraction with the swin transformer network followed by cancer detection using ensemble learning, obtaining a superior accuracy of 99.44% for colposcopy images compared to existing models (48).

By using clinical data and colposcopy images (49), developed a multimodal DL model for CC prediction (CIN2+ status). According to this study, the multimodal model surpassed clinician’s colposcopic impressions and showed better performance prediction of CIN2+(AUC-ROC = 95.3%, accuracy = 90.8%, PPV = 94.1%, NPV = 87.9%) and better calibration when compared to models that have used image or clinical history data alone (49). Summary of the studies reporting the use of AI-based models for cervical cancer diagnosis is shown in Table 1, including models presented later in section *6.0 Challenges and limitations in using AI*.

**TABLE 1:** Studies reporting the use of AI-based models in the screening and diagnosis of cervical cancer.

S. no.	Images or datasets included	AI model or algorithm developed/Used	Purpose of development	Quantitative results	Reference
1	VIA test	AI-based android application	Cervical pre-cancerous lesion detection	Sensitivity 80%, specificity 96.4%, accuracy 93.8%, precision 80%, ROC/AUC 0.85 (95% CI 0.66-1.0)	(31)
2	Cervigram images	Modified deep learning architecture CervixNet	Detection and classification of the cervigram images	IMODT database: Sensitivity 98.69%, specificity 98.76%, accuracy 99.27%. Guanacaste database: Sensitivity 99.22%, specificity 99.03%, accuracy 99.01%	(32)
3	Liquid-based cytology images	Deep learning model	Detecting CIN2	DL model vs. senior cytopathologist: Sensitivity (0.878 vs. 0.854), specificity (0.831 vs. 0.901)	(33)
4	Pap smear images	Deep transfer learning model for comparison of 16 pre-trained models	CC classification	Accuracy: Using herlev dataset: ResNet50 95% for 2-class and for 7-class classification. Using sipakmed dataset: VGG16 99.95% for 2-class and 5-class classification, DenseNet121 97.65% for 3-class classification	(34)
5	Pap smear images	Deep learning based dense CapsNet with Seg-UNet and denoising autoencoders	Classification of CC	Accuracy 99.65%	(35)
6	Cervical images from multiple dataset	Deep learning approach by integrating neural feature extraction and AutoInt models	Classification of CC images	Accuracy: KNN 99.96%, LGBM 99.92%	(36)
7	Liquid-based cytology images	Deep learning model	Identification and classification of CC	Accuracy 99.9%, sensitivity 99.8%, specificity 99.9%	(37)
8	Pap smear images	Deep ensemble learning by including CNN, AlexNet, and SqueezeNet	Squamous cell classification	Accuracy: CNN 90.8%, AlexNet 92%, SqueezeNet 91%, ensemble technique 94%	(38)
9	Pap smear images	Deep learning-based two-stream decision fusion network	Cervical pap-smear image classification	-	(39)
10	Whole slide cytology images	Deep learning-based compound scaling hypergraph neural network model (CSHG-CervixNet)	Classification of CC subtypes	Accuracy 99.31%, precision 98.97%, F1-score 99.34%	(40)
11	Pap smear images	Convolutional neural network (CNN) based model	Classification of CC cells	Accuracy: SIPaKMeD dataset: WSI patches (96.74% and full-cell images 97.55%. Herlev dataset: 90.42%	(41)
12	H&E-stained images	Deep learning based Silva3-AI model	Silva pattern-based classification of endocervical adenocarcinoma	AUROC: Silva C 0.947, silva B 0.908, silva a 0.947	(45)
13	Pap smear test WSI	Self-supervised learning methods	Classification of WSI of pap smear test	-	(42)
14	Colposcopy images	Deep learning model	Classification of cervical transformation zone	Accuracy 88.49%, sensitivity 85.95%–90.12%	(47)
15	Colposcopy images	LSTEDL	Detect and classification of colposcopy images	Accuracy 99.44%	(48)

## 4 AI in cervical cancer prognosis

Prognosis is the likely outcome or course of a disease. The prognosis of CC depends on several factors like cancer stage at the time of diagnosis (size of tumor, spread to other parts of body or involvement of lymph nodes), type of cancer (squamous cell carcinoma (SCC) or adenocarcinoma (AC)), response to treatment, patient’s general health, presence of co-morbidities, whether the cancer is newly diagnosed or recurred (50, 51). AI-based models played a significant role in the prognosis of CC by predicting survival outcome, lymph node metastasis, treatment outcome and post-operative risk factors.

### 4.1 Predicting survival outcome by using AI-based tools

Staging of CC (by FIGO) is important to predict the prognosis or plan for the treatment (52). FIGO, categorizes CC into different stages, ranging from 0 to IV, with higher stages indicating more advanced disease (53). Early-stage CC diagnosis has higher 5-year survival rate (91%) compared to later stages (60 or 19% depending on the spread to near/distant parts of the body).

Using surveillance, epidemiology and end results (SEER) database (54) developed a personalized survival prediction DL model for patients with CC. Authors reported that model out-performed four other survival prediction models and offered more precise prognostic information than conventional FIGO stages (54). Another study found that a DL model based on clinical risk factors and pathological features could accurately predict the 5-year survival outcome for non-surgical CC patients receiving radiotherapy (55).

Similarly, (56) developed a DL‐based pathological risk score (RS) to predict patients’ prognosis. The RS model, which applied a CNN with an autoencoder to extract features from clinical characteristics, was reported to effectively predict prognosis and stratify those results data (56).

In order to predict disease-free survival (DFS) in patients with locally advanced cervical cancer (LACC) after definitive radiotherapy, Wang, Weiping, et al. 2025 created CerviPro, a DL-based multimodal prognostic model by integrating clinical variables, handcrafted radiomic features, pre- and post-treatment CT imaging. Multimodal feature fusion consistently outperformed models based on single feature categories (clinical data, imaging, or radiomics alone), demonstrating the synergistic value of merging several data sources.

### 4.2 Predicting lymph node metastasis (LNM) by AI-driven models

The presence of LNM has a direct impact on patients’ prognosis and is a major determinant of treatment selection and prognosis (57). Surgical histopathology remains the standard method for LNM diagnosis, highlighting the need for non-invasive AI diagnostic techniques (58). (58) developed a ranking attention multiple instance learning (RA‐MIL) model to diagnose LNM in CC patients using MRI images (58). Authors highlighted promising performance and improvements compared to other DL models (58). In similar study, (59) developed a multi-instance deep CNN based on a multiscale attention mechanism. The study reported that the network accurately predicted preoperative lymph node status of patients with CC based on H&E stained WSIs of primary cervical tumors (59).

It is challenging to predict normal-sized LNM in CC, so (60) evaluated feasibility of an AI solution by developing a non-invasive DL nomogram model based on readout segmentation of long variable echo-trains diffusion weighted imaging (RESOLVE-DWI) using MRI images and clinical data (60). In another study, the investigators developed a DL model using the Vision Transformer (ViT) and recurrent neural network (RNN) frameworks to predict LNM based on pre-operatively obtained histopathological WSIs of primary CC patients (57). Similarly, (61) developed a DL‐based nomogram for predicting LNM in CC patients using CT and validated it in a large‐scale, multicenter dataset, surpassing diagnostic performance of experienced gynecologists (61). Authors of these studies reported potential of DL-based models to aid LNM determination and treatment decision-making (57, 61). However, these studies require further external validation with larger datasets for clinical application.

### 4.3 Predicting treatment response by DL models

Concurrent chemoradiotherapy is the standard of care for locally advanced CC, but treatment efficacy varies significantly among patients. To address this, (62) sought to predict CRT responses by developing prediction models based on pretreatment MRI data (62). For some CC patients, radical surgery after neoadjuvant chemoradiotherapy is the preferred treatment over CRT (63). (63) developed a similar treatment prediction model (MRI-based), using radiomics and DL, focused on the response to NACRT (63). While both studies emphasize AI’s potential to aid clinicians in personalizing treatment and predicting survival, they require further external validation using larger datasets before clinical implementation (62).

### 4.4 Predicting post-operative risk factors by ML model systems

Radical hysterectomy is another common treatment for early-stage CC, so (64) developed a ML model to predict post-operative risk (64). The proposed ML model provides critical information on pathologic risk factors prior to surgical intervention (64).

### 4.5 Predicting recurrence after radiotherapy by DL models

Reliable biomarkers that can identify patients at high risk of recurrence are necessary, as one-third of patients experience recurrence following radiotherapy. Using MRI scans, (65) created a GANs-based image synthesis model to predict recurrence following radiation treatment in locally advanced cervical cancer. GANs are powerful DL models that generate realistic, new data that mimics a training dataset by using two competing neural networks against each other: a generator and a discriminator. According to the study, the synthesized MR image’s prediction ability was equivalent to the actual MR image (65).

A summary of the studies evaluating AI-based models for the prognosis of CC is shown in Table 2.

**TABLE 2:** Studies reporting use of AI-based models in the prognosis of cervical cancer.

S. no.	Images or datasets included	AI model or algorithm developed/Used	Purpose of development	Quantitative results	Reference
1	Cervical adenocarcinoma patients from SEER database	DL model	Personalized survival prediction	DL model reached 0.878 c-index and 0.09 brier score in the test set and 0.80 c-index and 0.13 brier score in the external test set	(54)
2	Clinical features and HE-stained pathologic images from non-surgical CC patients treated with radio-chemotherapy	DL model	Predict 5-year overall survival	Clinical-pathomic model AUC 0.750, the clinical predictor model AUC 0.729, and pathomic model AUC 0.703	(55)
3	Clinical characteristics and WSI from stage IA1–IIA2 CC patients who underwent surgery	DL‐based pathological risk score (RS)	Predict survival in CC	C-index 0.700, AUC 0.800	(56)
4	Histopathological whole‐slide biopsy images	Weakly supervised DL model	Lymph node metastasis prediction	Internal test set: AUC 0.919, accuracy 0.909. In prospective cohort: AUC 0.91, ACC 0.895	(57)
5	T2‐weighted imaging (T2) MRI images of CC patients	RA‐MIL model	Lymph node metastasis prediction	AUC of internal test set: 0.809, and public dataset: 0.833	(58)
6	H&E stained WSI of primary cervical tumors	Deep CNN based framework	Lymph node metastasis prediction	AUC of cross-validation set is 0.87, internal independent test set is 0.84, external test set is 0.75	(59)
7	Dataset of MR images and patient information	RESOLVE-DWI-based DL nomogram	Normal-size lymph node metastasis prediction	Multi-channel CNN model: development cohort (AUC 0.848) and test cohort (AUC 0.767). DL nomogram: development cohort (AUC 0.890) and test cohort (AUC 0.844)	(60)
8	CT images	DL‐based nomogram	Lymph node metastasis prediction	AUC: training cohort is 0.867, validation cohort is 0.807, external testing cohort-1 is 0.781, and external testing cohort-2 is 0.804	(61)
9	T1 and T2-weighted MRI images	Comparing DL and handcrafted radiomics	Predict treatment response	Using only MRI data: DLR AUC 0.721, HCR AUC 0.597. Integration of clinical factors into the MRI data: DLR AUC 0.782, HCR AUC 0.676	(62)
10	T1 and T2-weighted MRI images	Multimodal MRI-based deep-radiomics model	Predict treatment response	Hybrid feature set: AUC 0.86, accuracy 0.75, precision 0.81	(63)
11	Radical hysterectomy patients	Machine learning model	Predict postoperative pathologic risk factors	Best predictive performance was shown by: Random forest for DSI (70.8% accuracy, 0.767 AUC), cforest for LNM (64.3% accuracy, 0.620 AUC), and elastic net for LVSI (59.7% accuracy, 0.628 AUC)	(64)

## 5 AI in cervical cancer treatment

The CC treatment options vary based on the stage of the cancer and the patient’s overall health (66). Early-stage CC is often treated with surgery or radiation, sometimes combined with chemotherapy. Advanced stages often involve radiation therapy with chemotherapy or chemotherapy alone (66, 67).

Radiation treatment of CC requires effective dose prediction and treatment planning to ensure maximum radiation to maximize tumor dose and minimize healthy tissue exposure. Techniques such as brachytherapy, intensity-modulated radiation therapy (IMRT), and volumetric modulated arc therapy (VMAT) are employed for dose delivery (68). In recent years, AI has fundamentally transformed radiotherapy workflows, enabling automated target delineation, precise organ-at-risk segmentation, efficient dose prediction, and optimized treatment planning. In recent years, AI has played a significant role in dose prediction, treatment planning, target volume identification and organ at risk determination.

### 5.1 Segmentation of clinical target volume (CTV) and organs at risk (OAR)

In radiation therapy, the CTV is the tissue volume that needs to be treated, including the gross tumor volume (GTV) and any potential microscopic spread of cancer (69). OAR are the healthy tissues near the CTV that could be damaged by radiation. Imaging techniques like MRI, PET and CT play a crucial role in determining the CTV and OAR (70).

DL-based segmentation networks can offer efficiency by improving the GTV delineation process and reducing manual segmentation. Many DL-based solutions rely on traditional CNN frameworks, including the CNN-based image segmentation model U-Net and its variants (71). Rodríguez Outeiral, Roque, et al. 2023 developed a 3D nnU-Net model that achieved reasonable segmentation of GTV on brachytherapy MRI images, although there was high variability among patients (72). Huang, (71) developed a similar framework using an improved M-Net model (M-net_IM), another variant of U-Net (71). This model relied on CT images rather than MRI and outperformed the selected comparative models (71).

Beyond standard DL-based models that rely on conventional CNN or U-Net variants, researchers have developed improved segmentation models. (73) developed a dual convolution-transformer UNet (DCT-UNet) model as a hybrid model. Incorporation of CNN and transformer features allows the model to achieve both fine and coarse segmentation of OAR and CTV from female pelvic MRI, enabling fast and accurate automatic segmentation (73). Separately, Tian, Miao, et al. 2023 developed a parallel-path attention fusion network (PPAF-net) to accurately delineating CTV and OAR from CT data (74). The Multi-Decoder and Semi-Supervised Learning (MDSSL) model developed by Peng et al. 2024 significantly improved cervical cancer CTV auto-segmentation using limited CT data (75).

Though many auto-segmentation programs are based on CNNs and U-Net architecture, CNNs are limited due to their local focus and reduced global context (76). To address this issue, (76) introduced the AM-UNet, an automatic segmentation model leveraging the Mamba state-space model, demonstrating significant consistency between automatic and manual segmentation of high-risk CTV and OAR in CC brachytherapy (76).

Transfer learning (TL) is a machine learning technique that uses information from one task or dataset to enhance model performance on a different dataset or related task. Variations across institutions and clinical settings prompted (77) to explore adaptable models by exploring the effectiveness of TL to improve the generalizability of DL models for auto-segmentation of OAR (77). The authors reported that transfer learning can serve this function in MR-guided CC BT (77).

In order to improve the accuracy of automatic contouring organs at risk during cervical cancer radiation therapy, (78) developed a deep reinforcement learning (DRL) method, which incorporates the segment anything model (SAM). In order to automatically delineate OAR, DL is utilized to extract graphic features from high-dimensional raw CT data, and RL is used to learn through interactions with the results of manual adjustments made by physicians, maximizing the cumulative rewards.

The DRL model out-performed the DL model, suggesting that combining SAM and RL increases segmentation accuracy and consistency in comparison to traditional DL techniques. This introduces a novel training strategy, which optimises performance without increasing complexity to the model (78).

### 5.2 Dose prediction

Relying on the same CNN architecture, a 3D-Unet dosage prediction model was developed by Qilin, Zhang, et al. in 2022 to accurately predict the 3D dose distribution of VMAT for CC (79). This research explored model generalization, an important feasibility metric for dose prediction. Similarly, in order to predict the voxel-based VMAT dosage distribution for postoperative CC patients, (80) adapted and evaluated two DL network models (3DUnet and 3DResUnet) (80). This presents a novel training approach that optimises performance without making the model more complex (80).

(81) developed a 3D deep CNN model (RapidBrachyDL), to address the challenges from current dose calculations methods, which are time-consuming (81). The study focused on increasing speed of dose predictions and reported that RapidBrachyDL accurately estimated dosage for patient-specific brachytherapy dosimetry (81).

Gautam, (82) incorporated an attention-gating mechanism into their model, which is in addition to 3D UNET architecture for treatment planning in CC high-dose-rate brachytherapy (HDRBT) (82). Results of this study reported that attention-gated 3D-UNET model demonstrated greater capability in predicting voxel-wise dose prediction, in comparison to 3D-UNET, for direction modulated brachytherapy and intracavitary brachytherapy planning (82).

Beyond CNNs, (83) developed a novel DL-based algorithm, AtTranNet, that utilized transformer-based architecture to incorporate attention gating and long-range capabilities (83). AtTranNet was evaluated for three-dimensional dose prediction of CC VMAT in multicenter datasets, and study authors reported that the algorithm accurately performed dose prediction for CC in multiple centers (83).

### 5.3 Treatment planning and verification

In addition to dose prediction, AI has also been applied to add efficiency across the treatment planning process. (84) developed an automatic IMRT program for cervical cancer that features a CNN-based prediction model and an automated optimization strategy (84). The study found that the IMRT plans produced superior dose sparing while maintaining target dose effectiveness (84). (85) developed a DL method that skips the time-consuming inverse optimization process for automatic generation of machine-deliverable IMRT plans that match clinical quality, enabling acceleration of the treatment planning process (85).

Treatment planning aids have also incorporated verification and accuracy components to ensure whether structures are meeting clinical needs. (86) developed a DL-based CNN auto-segmentation model that achieved agreement with most CC EBRT plannings structures (86). In another study, investigators have developed a DL-based strategy to predict dwell positions and times in HDRBT for independent plan verification (87). Taking this one step further, (88) developed an adversarial deep-learning framework, integrating a dose prediction network and a plan-approval probability network, to accurately predict the probability of plan approval for HDRBT in CC patients (88).

Summary of the studies reporting the use of AI-based models for CC treatment is shown in Table 3.

**TABLE 3:** Studies reporting use of AI-based models in the treatment of cervical cancer.

S. no.	Images or datasets included	AI model or algorithm developed/Used	Purpose of development	Quantitative results/Inference	Reference
1	CT images	Improved deep learning M-net model (Mnet_IM)	Segmentation of CTV in CC brachytherapy	Mnet_Im: Mean ASD 0.690, mean HD95 3.2013, mean SD 0.8084, mean SO 0.7785, mean VD 0.8828	(71)
2	T2-weighted (T2w) MRI images	Deep learning based automatic segmentation algorithms	Segmentation of the gross tumor volume (GTV)	Median dice 0.73, median 95th HD 6.8 mm and median MSD of 1.4 mm	(72)
3	MRI images	Deep learning based dual convolution-transformer UNet (DCT-UNet)	Automatic segmentation of high-risk CTV and OAR in CC high-dose-rate brachytherapy	DCT-UNet achieved mean dice similarity coefficient 0.932 for bladder, 0.786 for rectum, 0.663 for sigmoid colon, and 0.741 for HR-CTV	(73)
4	CT images	PPAF-net	Automatic delineation of CTV and OAR	DSC and hausdorff distance of 88.61% and 2.25 cm for CTV, 92.27% and 0.73 cm for rectum, 96.74% and 0.68 cm for bladder, 96.38% and 0.65 cm for left kidney	(74)
5	CT scan images	Auto-segmentation algorithm based on MDSSL	Determine the CC target volume	MDSSL model: Post-operative radiotherapy for CTV1: DSC 0.80, HD95 5.85 mm and ASD 0.95 mm, for CTV2: DSC 0.84, HD95 4.88 mm, ASD 0.73 mm	(75)
6	CT scan images	Automatic segmentation model based on AM-UNet	Segmentation of high-risk CTV and OAR in CC brachytherapy	AM-UNet achieved mean DSC of 0.862, 0.937, 0.823 and 0.725 for HRCTV, bladder, rectum, and sigmoid respectively	(76)
7	T2-weighted MRI image dataset	Transfer learning approach to enhance the generalizability of DL model	Auto-segmentation of OAR in cervical brachytherapy	Mixed model demonstrated superior performance on unseen data	(77)
8	VMAT plans	DL-based 3D‐Unet dose prediction model	Dose prediction of CC VMAT	Developed model can predict the dose distribution accurately	(79)
9	VMAT plans	DL networks 3DResUNet and 3DUNet	Predict the voxel-based dose distribution of VMAT for CC patients	Maximum dose difference was observed in D98 of rectum with a | δD| of 5.00% ± 3.40% and 4.88% ± 3.99% for Unet3D and ResUnet3D, respectively	(80)
10	CT images, contours of CTV and OAR, and treatment plan	Deep learning based 3-dimensional deep convolutional neural network (CNN) model RapidBrachyDL	Dose distribution prediction in high-dose-rate brachytherapy	RapidBrachyDL demonstrated good generalization to cervical data with 1.73% for CTV D90, 2.46% for rectum D2cc, 1.68% for sigmoid D2cc, and 1.74% for bladder D2cc	(81)
11	Brachytherapy plans	Attention‐gated 3D UNET	Dose distribution prediction of high-dose-rate brachytherapy of CC	Mean individual differences in ΔD2cc were 0.38 Gy for bladder, 0.43 Gy for rectum, and 0.47 Gy for sigmoid	(82)
12	VMAT plans	Deep learning based AtTranNet algorithm	Dose prediction of CC VMAT	The maximum |δD| for planning CTV was observed in D98 (1.24 ± 2.73 Gy). The maximum |δD| for OAR was observed in dmean of bladder (4.79 ± 3.14 Gy). The maximum |δV| were observed in V40 of pelvic bones (4.77% ± 4.48%)	(83)
13	Patients treated with IMRT	CNN based automated intensity modulated radiation therapy model	Dose prediction of CC IMRT	Automatic IMRT plans optimized from the CNN generated objectives have superior dose sparing without compromising of target dose	(84)
14	IMRT plans	Deep learning framework	Automatic generation of IMRT plan for CC radiation therapy	Deep learning framework can produce machine-deliverable IMRT plans with quality similar to the clinical plans in the test set.	(85)
15	EBRT plans	CNN based auto-segmentation model	Evaluate the accuracy of EBRT planning structures	DL based auto-segmentation model can achieve clinically acceptable contours for most of the EBRT planning structures in CC patients	(86)
16	HDRBT cervical patients	Deep inception network-based architecture	Treatment plan check and verification of high-dose rate brachytherapy	The model provides an efficient and accurate tool for independent verification of HDR brachytherapy treatment plan	(87)
17	HDRBT plan	DL framework consisting of dose prediction network and a plan-approval probability network	Predict the probability to approve high-dose-rate brachytherapy plan	Developed a novel DL framework that predicts a probability of plan approval for HDRBT of CCs	(88)

## 6 Integration of electronic health records (EHRs) with AI models

Integrating AI with EHRs creates intelligent systems that analyze vast patient data for better diagnosis, personalized treatment, predictive risk assessment through technologies like natural language processing (NLP), feature engineering and extraction, multimodal data fusion, predictive analytics, and clinical decision support systems (CDSS). Integrating AI with EHRs revolutionizes cervical cancer care by enhancing screening (automated Pap analysis, HPV detection), improving diagnosis (faster, more accurate image/pathology review, biomarker identification), refining prognosis (predicting recurrence/survival with multi-modal data), and personalizing treatment (tailored plans, optimizing drug response) through analyzing vast patient data, boosting efficiency, and enabling precision oncology, despite data/ethical challenges (89). AI algorithms analyze Pap smears and histopathology images, improving accuracy and consistency in detecting abnormal cells, reducing human error. AI models identify high-risk HPV types and molecular markers from EHR data, allowing for better risk stratification and targeted follow-ups. Multimodal AI models enhance cervical cancer diagnostic and staging accuracy by integrating pathology reports with imaging findings like MRI and CT scans. AI models predict cancer risk and patient survival, guiding proactive management and personalized screening schedules. AI models analyze patient data (genetics, lifestyle, disease stage) to recommend optimal, individualized treatment regimens (chemo, radiation, targeted therapy).

## 7 Challenges and limitations in using AI

Despite substantial advancements in AI models for the screening, diagnosis, prognosis, and treatment of cervical cancer, several challenges and concerns impede the integration of these tools into clinical practice. Challenges of using AI-based tools includes: (a) *data related:* Having dataset of high-quality, low-bias and large volume. In addition, the complexity and imbalance in the available data sets, security and privacy policies do play important roles in the analysis of data and result output; (b) *model related*: Complexity of developed model systems, selection, training, generalizability, limited external validation, evaluation of model performance, lack of transparency, explainability, robustness, reproducibility, accountability, and scalability; (c) practical integration and interpretability in AI for CC: While this review synthesizes advances in AI across CC screening, diagnosis, prognostication, and therapeutic stratification, we acknowledge the importance of distinguishing theoretical innovation from clinically implemented systems. Many high-performing algorithms remain confined to experimental settings without prospective validation, regulatory authorization, or integration into routine clinical workflows.

For translational relevance, AI systems must extend beyond methodological innovation to demonstrate real-world feasibility, interoperability with laboratory and hospital infrastructure, and sustained performance across diverse populations. Furthermore, incorporation of interpretable modeling frameworks is essential to support clinician trust, regulatory evaluation, and ethical deployment. Addressing practical implementation and explainability is therefore fundamental to advancing AI from proof-of-concept research to clinically actionable tools in cervical cancer management.

### 7.1 Practical integration and real-world validation

Recent years have witnessed the transition of AI-assisted cytology and colposcopy systems from experimental frameworks to regulated clinical deployment. The Genius™ Digital Diagnostics System (Hologic, Inc.), which incorporates deep learning–based cervical cytology analysis, received FDA clearance for digital cytology screening and has been integrated into routine laboratory workflows in the United States. Prospective real-world validation has also been demonstrated in large population cohorts. The AICCS system, validated prospectively in 16,056 Chinese participants, delivered 89.2% accuracy (AUC 0.947) for CIN2+ detection, cutting colposcopy referrals by 33% in community clinics despite implementation challenges including staff training and data standardization (90). Similarly, population-based implementation studies in large screening cohorts have shown high concordance between AI-assisted and manual cytology interpretation, with increased sensitivity for high-grade lesions and preserved specificity.

Multicenter prospective trials further underscore operational feasibility. Ongoing clinical trial (e.g., NCT06644248 (https://clinicaltrials.gov/study/NCT06644248)) evaluating AI-assisted colposcopy and cytology platforms across diverse hospital settings, including LMICs, where workforce shortages amplify the value of automation. In deployed regional screening networks, AI-based systems have demonstrated high atypical cell detection rates and reductions in referral burden (44). Reported workflow efficiencies include substantial reductions in slide review time, supporting scalability in high-volume laboratories. Collectively, these studies move beyond theoretical modeling and demonstrate measurable clinical impact, regulatory oversight (e.g., FDA 510(k) clearance), and real-world feasibility.

### 7.2 Implementation strategies

Challenges pertaining to the implementation of developed AI tools include ethical and regulatory considerations, integration with clinical workflow, accountability, cost, public perspectives, education, training healthcare professionals and compliance are some of the points to be considered while deciding to use AI-enabled platforms (91–93).

Large volume, high-quality, labelled data sets are required for training, testing and validation of AI models. Acquiring such datasets for CC is very difficult due to limited access, security and patient privacy concerns (94, 95). Furthermore, models trained on biased datasets are likely to provide inaccurate predictions, which can amplify existing health disparities (96). Recent efforts to address these data-related concerns have included more robust data to make predictions. In order to investigate the synergistic benefit of integrating colposcopy, cytology and HPV test for improving cervical cancer screening performance (97) developed a colposcopy-based DL model. The study reported that the integrated DL model offered increased accuracy of cervical screening (97). Data complexity and dimensionality is another common challenge that makes it difficult to train comprehensive AI models and ensures interoperability (98, 99). The significance of integrating logical reasoning, inference, and discovery into diagnostic frameworks was recognized by Taghados, Zahra, et al. in 2025 (100). This approach goes beyond observational correlations to uncover hidden causal relationships to ensure comprehensibility (100). The investigators of this study developed CausalCervixNet, a CNN with Causal Insight (CICNN) that uses causality-based methodologies to more efficient and accurate CC cell classification (100).

The “black-box” nature of the AI systems especially DL models, where internal decision-making processes are hidden, presents a significant challenge to establishing accountability, trust, and ethical compliance (101). Without explainable AI (XAI), it can be difficult to trust model predictions. For example, factors such as the presence of mucus or blood, over and under exposure, poor light and focus, noise and blur hamper accurate detection of precancerous lesions or cancer (102). Without knowing explainable AI (XAI), it can be difficult to trust model predictions. To address this, Ahmed, Syed Rakin, et al. 2025 developed a model that classifies images of the cervix into “low,” “intermediate” and “high” quality categories (102). This tool offers a better understanding of how misclassification can occur.

AI models often perform well on the specific datasets they were trained and may not generalize well when applied to new datasets in different populations (103). Limited evaluation and external validation is likely to hinder the deployment of effective and reliable AI systems (104). Many of the studies explored in this review have included prediction performance evaluation in comparison to the performance of other models or trained gynecologists, dosimetrists and oncologists. Selection of the correct model and reproducibility of the results is very difficult particularly when same models are trained on different datasets (105). Further studies similar to Ni, Ruiyan, et al. 2024 are essential to increase the adaptability and generalizability of models (77).

Rapid AI advancement in healthcare demands robust ethical guidelines and regulatory frameworks to guarantee patient safety and responsible innovation (106). Integration of EHRs with AI models faces multiple challenges related to quality of the data (incompleteness, bias), technical hurdles (interoperability, system integration, costs), ethical/regulatory gaps (privacy, accountability, lack of global standards), transparency, trust (black box nature), adoption by healthcare professionals and clinical validation in diverse settings (107). Clinicians and patients need to trust and understand the AI models before adopting them in practice. Healthcare professionals should be educated and trained on how to successfully integrate AI tools into their workflows. AI should augment, not replace, human expertise in healthcare. To ensure safety and accuracy in screening, diagnosis, and treatment, active human oversight is necessary.

### 7.3 Explainability and model interpretability in cervical cancer AI

In oncologic decision-making, interpretability is essential for clinical trust, regulatory approval, and ethical deployment. Although deep learning systems demonstrate high diagnostic accuracy for cervical cytology, colposcopy, and histopathology, opaque “black-box” architectures may limit adoption in regulated clinical environments. Regulatory agencies increasingly require transparency, traceability, and post-market surveillance, reinforcing the need for explainable AI (XAI) in cervical cancer screening and diagnosis. Several XAI methodologies are now incorporated into cervical cancer modeling.

SHAP (SHapley Additive exPlanations) has emerged as a powerful XAI tool for dissecting feature contributions in CC prediction, particularly for HPV-driven risk stratification. SHAP was applied to enhance interpretability in a large retrospective CC risk prediction study utilizing routine hematologic parameters (108). In this cohort (2013–2023), 2,503 histopathologically confirmed CC cases were compared with 3,794 controls, including women with other gynecologic conditions and healthy individuals. Parameters like age, clinical information, and 22 peripheral blood indices were evaluated. Among four machine learning algorithms tested, the extreme gradient boosting (XGBoost) model achieved the highest discriminative performance (AUC = 0.964), whereas the random forest model demonstrated comparatively lower generalizability (AUC = 0.907) (108).

Importantly, SHAP-based attribution analysis ranked the top six contributors to CC occurrence and identified PDW as the most influential predictor. Visualization through SHAP summary and force plots enabled case-level explanation of risk contributions, demonstrating the relative influence of inflammatory and hematologic parameters in disease occurrence. This interpretability framework aligned predictive outputs with biologically plausible mechanisms particularly inflammation-related platelet dynamics thereby enhancing clinical transparency and supporting potential integration into risk-stratified screening strategies. Complementing this, a radiomics study in 268 CC patients (n = 185 for training; n = 83 for validation) extracted 124 T2-MRI features, refining 12 via ICC (>0.75), mRMR, and LASSO; logistic regression outperformed peers (validation AUC 0.818; combined model 0.844; P = 0.005 vs. clinical factors), with SHAP identifying tumor heterogeneity (original_firstorder_Minimum, GLCM-correlation) as pivotal for recurrence risk—net benefits confirmed by DCA, supporting personalized adjuvant therapy (109). Together, these applications demonstrate SHAP’s versatility across tabular hematologic and imaging data, boosting clinician trust and regulatory viability in multimodal cervical cancer AI.

Grad-CAM (Gradient-weighted Class Activation Mapping) enables precise spatial localization of discriminative regions in low-magnification cytology and colposcopy images, verifying that predictions prioritize biologically relevant features like nuclear atypia, koilocytosis, and epithelial irregularities over artifacts. In a Noisy Student Training study using EfficientNet on 140 whole-slide liquid-based cytology images (50 LSIL, 50 HSIL, 40 negative; yielding 56,996 patches), Grad-CAM heatmaps visualized abnormal cell clusters driving binary normal/abnormal classification (AUC 0.908, accuracy 0.873, F1 0.833), confirming focus on low-magnification abnormalities with optimal confidence thresholds and augmentations for resource-limited screening (110). Complementing this, the CASPNet model, integrating multi-head self-attention, cross-stage partial connections, and SPPF layers achieved 97.07% accuracy on the SIPAKMED benchmark for healthy vs. malignant cervical cells, leveraging attention to capture global context and multi-scale features for superior efficiency over standard CNNs (111). Attention-based architectures like CASPNet further boost transparency by dynamically weighting radiomic features and histopathologic patterns, balancing local extraction with global reasoning in constrained clinical settings. A study adapted ResNet via Grad-CAM for medical text classification, attaining recall 90.9%, precision 91.1%, and F1 90.2% over 25 epochs. The heatmaps generated intuitively highlighted decision-critical words, extending XAI from images to multimodal cervical data integration (110). Collectively, these Grad-CAM and attention-driven approaches deliver 20%–30% higher clinician confidence versus black-box models, minimizing diagnostic overrides, enhancing biopsy concordance, and meeting regulatory mandates for transparent, traceable AI in cervical cancer screening.

LIME (Local Interpretable Model-agnostic Explanations) enhances CC risk prediction by treating complex “black-box” models as interpretable locally, revealing key decision drivers. In an H2O AutoML framework with stacked autoencoders and Fisher Score selection on UCI datasets (n > 1,000), LIME unpacked top deep learning predictions (95.24% accuracy, AUC 98.10%, log loss 0.1747), generating heatmaps that highlighted patient-specific factors (such as age >45, multiple sexual partners, >5 years smoking, and hormonal contraceptive use) as dominant contributors at the optimal F1 threshold (0.517), with confusion matrix errors of 5.75% (negatives) and 2.59% (positives). Complementing this, a Kaggle-based Stacked Ensemble AutoML study applied LIME to explain class probabilities across indices, visualizing STD history and behavioral risks to demystify black-box outputs, outperforming manual ML pipelines in efficiency while enabling clinician verification on commodity hardware (Python 3.8/Jupyter). These LIME applications demonstrate local fidelity across tabular risk data, aligning algorithmic outputs with HPV cofactors, thereby supporting traceable risk stratification and reducing overrides in resource-constrained settings like India (112).

Attention mechanisms intrinsically prioritize salient regions in CC imaging, providing heatmap-based transparency without *post hoc* computation. An EfficientNet-B0 architecture augmented with Convolutional Block Attention Module (CBAM) on pre-processed Pap-smear datasets (resizing, Otsu thresholding, morphological operations, color normalization) achieved 82% accuracy, 80.6% sensitivity, 83.1% specificity, and AUC 0.87 by channel-wise weighting of nuclear atypia and koilocytosis, outperforming baselines in underserved environments (113). Similarly, a fuzzy distance-based CNN ensemble (SimpleCNN, InceptionV3, Xception ± attention) fused via Euclidean/Manhattan/cosine metrics on wavelet-denoised, CLAHE-enhanced Pap-smears reached 98.3% accuracy (94% raw), with attention layers dynamically emphasizing cell morphology changes amid noise, while fuzzy aggregation ensured robust adaptation across models without retraining (114). Complementing these, Cervix-AID-Net and related colposcopy models employed multi-head attention to capture precancerous acetowhite patterns in IARC datasets, boosting AUC >0.95 by focusing on transformation zone irregularities over artifacts. Collectively, attention-driven systems deliver 20%–30% higher clinician agreement versus opaque CNNs, minimizing false positives in imbalanced cytology, enhancing biopsy targeting, and fulfilling FDA/EMA traceability mandates for deployable CC diagnostics (115). In summary, SHAP, Grad-CAM, LIME, and attention mechanisms collectively transform black-box cervical cancer AI into transparent, clinician-trusted systems, achieving 82%–98% accuracies while elucidating HPV cofactors, atypia, and imaging artifacts across tabular, cytology, and colposcopy data. These XAI advances yield 20%–30% higher diagnostic confidence and regulatory compliance, enabling scalable early detection and personalized care in resource-limited settings.

## 8 Conclusion and future directions

**FIGURE 5:**
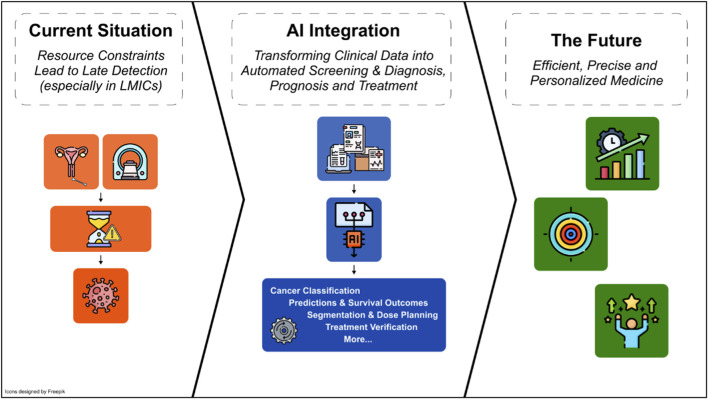
Summary of AI in the CC landscape.

In conclusion, we have summarized the recent advancements in application of AI-based approaches for screening, diagnosis, prognosis and treatment of CC. A visual summary of these efforts is depicted in Figure 5. AI algorithms, particularly ML and DL, have played a significant role in transforming CC screening, diagnosis, prognosis and treatment by outperforming human experts. AI-powered tools can analyze digitalized cytological, histopathological, colposcopic images to detect abnormal cells or lesions and contribute to fast, accurate and early detection of CC. AI-based prognostic models, particularly DL models, integrated clinical, histopathological, radiomic data to predict LNM, treatment response, survival outcome, and post-operative risk factors, thereby contributing to better patient outcomes. By analyzing advanced radiological images and treatment plans, AI-based models are transforming CC treatment by segmentation of CTV, OAR, dose prediction and treatment planning.

The integration of multimodal data sets, including clinical variables, imaging, genomic, proteomic, and patient-reported outcomes, facilitates a thorough evaluation that enhances diagnostic accuracy and accelerates treatment initiation, ultimately improving patient outcomes. Integration of complementary information from different data sources improves diagnostic accuracy, risk stratification and personalized treatment recommendations. Collaboration across multiple centers and institutions is required to generate large, high-quality and diverse datasets for training, testing, validating and generalizability of the AI models. The advancement of explainable and transparent AI is essential for comprehending the decision-making processes of AI models and building trust among clinicians. Ethical and regulatory guidelines should be developed by engaging patients, healthcare providers, policymakers and regulatory agencies by considering data security, patient privacy, informed consent and algorithm bias.
